# Mesenchymal Stem Cell-Derived Neuron-Like Cell Transplantation Combined with Electroacupuncture Improves Synaptic Plasticity in Rats with Intracerebral Hemorrhage via mTOR/p70S6K Signaling

**DOI:** 10.1155/2022/6450527

**Published:** 2022-02-15

**Authors:** Guoqiang Yang, Jiayi Zhu, Guwen Zhan, Guangbi Fan, Li Deng, Huajun Tang, Xiaoqian Jiang, Bo Chen, Chaoxian Yang

**Affiliations:** ^1^Research Center of Combine Traditional Chinese and Western Medicine, Affiliated Traditional Medicine Hospital, Southwest Medical University, Luzhou 646000, China; ^2^Research Unit of Molecular Imaging Probes, Department of Radiologic Technology, Faculty of Associated Medical Sciences, Chiang Mai University, Chiang Mai 50200, Thailand; ^3^Department of Anatomy and Histoembryology, School of Basic Medical Science, Southwest Medical University, Luzhou 646000, China; ^4^Department of Emergency, The Fourth People's Hospital of Taizhou City, Taizhou 225300, China

## Abstract

Previous studies have shown that the combination of mesenchymal stem cell (MSC) transplantation and electroacupuncture (EA) stimulation is a neuroprotective strategy for treating intracerebral hemorrhage (ICH). However, the underlying mechanisms by which the combined treatment promotes neuroprotection remain unclear. This study was designed to investigate the effects of the combined treatment on synaptic plasticity and elucidate their underlying mechanisms. Therefore, rat ICH models were established by injecting collagenase and heparin, and the animals were randomly divided into model control (MC), EA stimulation (EA), MSC-derived neuron-like cell transplantation (MSC-dNLCs), and MSC-dNLC transplantation combined with EA stimulation (MSC-dNLCs+EA) groups. We observed the ultrastructure of the brain and measured the brain water content (BWC) and the levels of the microtubule-associated protein 2 (MAP2), galactocerebrosidase (GALC), and glial fibrillary acidic protein (GFAP) proteins. We also measured the levels of the phosphorylated mammalian target of rapamycin (mTOR) and 70 kDa ribosomal protein S6 kinase (p70S6K) proteins, as well as the expression of synapse-related proteins. The BWC increased in rats after ICH and decreased significantly in ICH rats treated with MSC-dNLC transplantation, EA stimulation, or combined therapy. Meanwhile, after ICH, the number of blood vessels increased more evidently, but only the combined treatment reduced the number of blood vessels among rats receiving the three treatments. Moreover, the levels of MAP2, GALC, postsynaptic density 95 (PSD95), and synaptophysin (SYP) proteins, as well as the levels of the phosphorylated mTOR and p70S6k proteins, increased in the MSC-dNLCs+EA group compared with those in the MSC-dNLCs and EA groups. Compared with the MC group, GFAP expression was significantly reduced in the MSC-dNLCs, EA, and MSC-dNLCs+EA groups, but the differences among the three treatment groups were not significant. In addition, the number of synapses increased only in the MSC-dNLCs+EA group compared to the MC group. Based on these data, the combination of MSC-dNLC transplantation and EA stimulation exerts a synergistic effect on improving the consequences of ICH by relieving cerebral edema and glial scarring, promoting the survival of neurons and oligodendrocytes, and activating mTOR/p70S6K signaling to enhance synaptic plasticity.

## 1. Introduction

Intracerebral hemorrhage (ICH) is a crucial cause of neurological morbidity and mortality worldwide [1–3] and is estimated to affect over 1 million people worldwide each year [4]. After ICH, hematoma or edema induces oxidative stress and the inflammatory response, excitotoxicity, and reactive oxygen species (ROS) generation, which may induce the death of a large number of neuronal cells. More than 30% of ICH survivors live with severe movement dysfunction, and over 70% of these patients suffer cognitive impairment [5, 6], but few proven treatments are used in clinical practice [2, 7–9].

Mesenchymal stem cells (MSCs) are considered promising seed cells for nervous system diseases because they have the properties of weak immunogenicity, good safety, and easy cultivation [10–12]. Many studies have confirmed that MSCs improve neurological functional recovery following ICH [13–17]. However, transplanted MSCs display a limited ability to repair damaged tissue because they do not substantially increase synapse-related protein expression in the damaged brain [18, 19]. Electroacupuncture (EA) is a part of the traditional Chinese medicine field. Some studies have shown that EA improves nerve function in the brain of subjects with ICH [20–23]. Does the transplantation of MSCs combined with EA therapy exert a good therapeutic effect? Previous studies have indicated that the transplantation of MSCs combined with EA stimulation promotes axonal regeneration and functional recovery of the injured spinal cord [24, 25] and leads to a better therapeutic effect by increasing the expression of neurotrophic factors, regulating neurogenesis, and increasing the neural differentiation of transplanted cells in ischemic stroke compared with a single therapy [26, 27]. We also confirmed that the combined treatment improves neurological function in rats with ICH in a previous study [28]. However, the exact mechanism by which MSCs combined with EA improve neurological function remains to be further explored.

After ICH, the destroyed brain structure induces the destruction of the synaptic structure. Increasing synaptic plasticity and rebuilding neural functional networks are the basis of restoring neural function. Synaptic plasticity is the ability of neurons to modify their connections and is involved in brain network remodeling following brain damage [29]. Although synaptic plasticity has been widely clarified in many other diseases, including Alzheimer's disease [30] and mood disorders [31], it has rarely been studied in hemorrhagic brain injury. The mammalian target of rapamycin (mTOR) signaling pathway senses and integrates various environmental signals to regulate organismal growth and homeostasis and regulates important cellular processes, including proliferation, growth, survival, and mobility [32]. Moreover, activated mTOR increases the levels of synaptic signaling proteins and increases the number and function of new spine synapses in depressed rats [33]. mTOR promotes protein synthesis by phosphorylating two key effectors, one of which is 70 kDa ribosomal protein S6 kinase (p70S6K) [34]. To date, studies have seldom focused on changes in mTOR/p70S6K signaling related to synaptic plasticity after ICH.

In this experiment, we examined the effects of a combined treatment with MSC-derived neuron-like cells (MSC-dNLCs) and EA on the brain water content (BWC), numbers of blood vessels and synapses, expression of marker proteins of neurons, oligodendrocytes and astrocytes, synapse-associated proteins, and levels of phosphorylated mTOR and p70S6K proteins in rats with ICH. We also investigated the effect of the combined treatment on synaptic plasticity and possible mechanisms.

## 2. Materials and Methods

### 2.1. Animals

**Figure 1:**
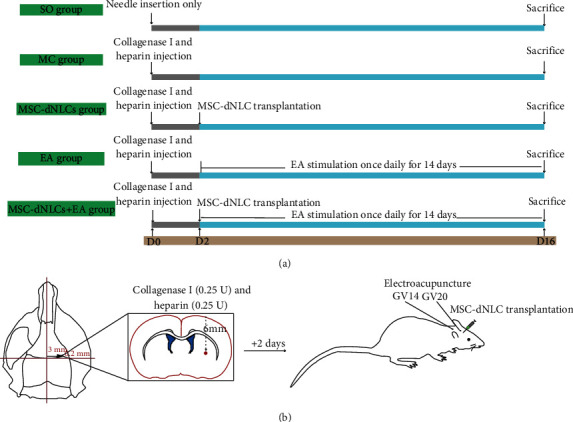
Conceptual illustrations of the experimental protocols. (a) Grouping and treatment strategies for experimental animals and brief timelines of the experimental procedures. (b) Schematic diagrams of the rat ICH model along with MSC-dNLC transplantation and EA stimulation.

Healthy adult Sprague–Dawley rats weighing between 200 and 250 g were provided by the SPF Laboratory Animal Center of Southwest Medical University (Luzhou, Sichuan, China). All of them were housed in the same animal care facility with a standard temperature (23 ± 2°C), lighting (12/12 h light/dark cycle), and relative humidity (65 ± 5%) and free access to food and water. The procedures for the animal experiments were performed in accordance with the Guidance and Suggestions for the Care and Use of Laboratory Animals formulated by the Ministry of Science and Technology of China. The animal protocol was approved by the Animal Ethics Committee of the Animal Center of Southwest Medical University (Luzhou, Sichuan, China), and the experimental procedures were optimized to minimize the number of animals and alleviate the pain experienced by the experimental animals. The rats were randomly divided into five groups: the sham operation (SO) group, the model control (MC) group, the MSC-dNLC transplantation (MSC-dNLCs) group, the EA stimulation (EA) group, and the combined treatment with MSC-dNLC transplantation and EA stimulation (MSC-dNLCs+EA) group, which were assigned to the following five experimental procedures, respectively (Figure 1(a)).

### 2.2. Rat Model of Intracerebral Hemorrhage

Rats were anesthetized by intraperitoneally injecting a dose of 40 mg/kg of 1% pentobarbital sodium and fixed on a stereotaxic apparatus (in a prone position) while maintaining the anterior and posterior fontanel**le**s at the same level. The scalp was incised sagittally approximately 10 mm; then, the anterior fontanelle was exposed after treatment with 30% H_2_O_2_. A burr hole (1 mm) was drilled on the right calvarial bone at a point 3 mm lateral and 0.2 mm anterior to the anterior fontanelle. A mixture containing heparin (1 *μ*l, 2.0 U/*μ*l) and collagenase I (2 *μ*l, 0.125 U/*μ*l) was drawn into a 5 *μ*l microsyringe. The needle was fixed on the stereotaxic apparatus and inserted into the caudate nucleus (location: 6 mm depth to the hole); the location of the injection point is shown in the schematic diagrams (Figure 1(b)). Then, the mixture was slowly injected. After injection, the burr hole was sealed with bone wax, and the skin was sutured. The sham group underwent the same procedure as described above, except that the mixture was not injected.

### 2.3. EA Stimulation

EA stimulation was conducted as described in our previous study [20]. The rats in the MSC-dNLCs+EA and EA groups were stimulated with EA 48 hours after successful modeling. Two sterile acupuncture needles with diameters of 0.25 mm were inserted into the Baihui (GV20) and Dazhui (CV14) acupoints and connected to the type G-6805 EA stimulator (HM6805, China). The rats were stimulated with continuous waves at a current of 1 mA, frequency of 3 Hz, and stimulation duration of 10 min once a day. EA was performed consecutively for fourteen days until the rats were sacrificed.

### 2.4. Resuscitation and Culture of MSCs

Rat MSCs labeled with green fluorescent protein (GFP) (Cyagen Biosciences, China) [28] were removed from liquid nitrogen, thawed in a 37°C water bath, and then centrifuged. After centrifugation, MSCs were resuspended in alpha-minimum essential medium (*α*-MEM) (HyClone) supplemented with 10% fetal bovine serum (FBS, HyClone), 100 mg/ml streptomycin, and 100 U/ml penicillin and incubated in a humidified atmosphere with 5% CO_2_ at 37°C. When the MSCs grew to 80% confluence, the cells were trypsinized using 0.25% trypsin and 1 mM EDTA and passaged. MSCs at passages 3-6 were used for subsequent experiments.

### 2.5. MSC Transplantation

Before transplantation, MSCs were induced as described in our previous study [35]. In brief, MSCs were treated with preinduction medium composed of *α*-MEM supplemented with 10% FBS and 1 mmol/l *β*-mercaptoethanol (*β*-ME) for 24 hours and then with neuronal induction medium composed of *α*-MEM supplemented with 1 mmol/l *β*-ME, 2% dimethylsulfoxide (DMSO), and 1 *μ*mol/l all-trans retinoic acid (RA) (Sigma) for 6 hours. Afterward, MSC-dNLCs were collected, and the cell density was adjusted to 2.5 × 10^7^ cells per ml. Then, an MSC-dNLC suspension (20 *μ*l) was extracted with a microsyringe and injected into the brains (location: right of anterior fontanelle: 3 mm; anterior of anterior fontanelle: 0.2 mm; depth: 6 mm) of rats in the MSC-dNLCs+EA and the MSC-dNLCs groups at an injection rate of 2 *μ*l/min after 48 hours when the rats were successfully modeled. Then, the needle hole was sealed with bone wax, and the skin was sutured and disinfected.

### 2.6. Brain Edema Examination

The BWC was measured using a previously reported method [36]. Briefly, animals were anesthetized and then decapitated. The brains were quickly removed, and the right basal ganglia were separated and weighed immediately on a precise electronic balance to determine the wet weight. After drying in an oven for 24 h at 100°C, the right basal ganglia were weighed again to measure the dry weight. The BWC was calculated as [(wet weight–dry weight)/wet weight] × 100%.

### 2.7. Immunohistochemistry

Rats were anesthetized by administering an intraperitoneal injection of an overdose of pentobarbital sodium and successively transcardially perfused with 0.9% normal saline and 4% paraformaldehyde in 0.01 M phosphate-buffered saline (PBS, pH 7.4). Subsequently, the brains were removed, postfixed, and dehydrated before frozen sections were cut at a thickness of 10 *μ*m with a freezing microtome (CM1950, Leica, Germany).

After permeabilization with 0.3% Triton X-100 in 0.01 M PBS for 30 min at room temperature (RT) and blocking with 10% goat serum, sections were immunostained with the following primary antibodies: rabbit anti-laminin (Boster, China, diluted 1 : 100), mouse anti-MAP2 (Santa Cruz, USA, diluted 1 : 200), rabbit anti-GALC (Invitrogen, USA, diluted 1 : 200), mouse anti-GFAP (Santa Cruz, USA, diluted 1 : 200), rabbit anti-mTOR (CST, USA, diluted 1 : 200), mouse anti-p70S6K (Santa Cruz, USA, diluted 1 : 200), mouse anti-PSD95 (Santa Cruz, USA, diluted 1 : 200), and mouse anti-synaptophysin (SYP) (Santa Cruz, USA, diluted 1 : 200), which were diluted with 1% BSA/PBS (*w*/*v*). The sections were incubated with the primary antibodies overnight at 4°C and then incubated with horseradish peroxidase- (HRP-) conjugated goat anti-rabbit or anti-mouse secondary antibodies (Invitrogen, USA) at RT for 1.5 h. The sections were sequentially stained with diaminobenzidine and hematoxylin. Finally, the slices were imaged using a microscope (Olympus, Japan).

### 2.8. Immunofluorescence Staining

Frozen sections were washed with 0.01 M PBS for 10 minutes and then treated with 0.3% Triton X-100 at RT for 20 minutes. After blocking with 10% normal goat serum at 37°C for 30 minutes, the samples were incubated with primary antibodies (MAP2, GALC, GFAP, PSD95, and SYP) overnight at 4°C under humidified conditions and then incubated with Alexa Fluor 594-conjugated goat anti-mouse/rabbit IgG (1 : 500, Invitrogen) at RT for 60 min. Finally, sections were stained with 4′,6-diamidino-2-phenylindole (DAPI) and covered with fluorescence mounting medium (Dako).

### 2.9. Western Blotting

After anesthetization, the animals were decapitated, and brain tissues collected from rats in different groups were homogenized in ice-cold protein extraction reagent. Then, the total protein concentration in every sample from different groups was quantified using the BCA protein assay. Equal amounts of protein (50 *μ*g) were separated by sodium dodecyl sulfate–polyacrylamide gel electrophoresis and transferred to polyvinylidene fluoride (PVDF) membranes. Then, the membranes were incubated with primary antibodies against MAP2 (diluted 1 : 1000), GALC (diluted 1 : 1000), GFAP (diluted 1 : 1000), mTOR, p-mTOR (CST, USA, diluted 1 : 1000), p70S6K, p-p70S6K (Santa Cruz, USA, diluted 1 : 1000), PSD95 (diluted 1 : 1000), SYP (1 : 1000), and GAPDH (Abcam, United Kingdom, diluted 1 : 10000) at 4°C overnight. After incubation with HRP-conjugated goat anti-mouse/rabbit IgG (Bio-Rad, USA, diluted 1 : 2000) at RT for 2 h, the membranes were immersed in an enhanced chemiluminescence (ECL) solution (Millipore, USA) and exposed using an Image-Quant ECL Imager. The protein levels were normalized to the corresponding amount of GAPDH and analyzed using Quantity One software.

### 2.10. Ultrastructural Observation

Rats were anesthetized, their brains were removed, and caudoputamen samples (the peripheral hematoma zone) were dissected into 1 mm^3^ pieces and fixed with 3% glutaraldehyde at 4°C. After fixation with 1% osmium tetroxide for 2 h, the pieces were dehydrated through a graded series of acetone solutions, infiltrated with propylene epoxide, and embedded in Epon 618 resin. Sections were cut at a thickness of 40 nm, mounted onto a 200-mesh copper grid, and observed with a JEM-1400 series transmission electron microscope (TEM) (Japan Electron Optics Laboratory, Japan). Digital images of the specimens were acquired using an integrated high-sensitivity complementary metal-oxide-semiconductor (CMOS) camera and analyzed by experienced electron microscopists.

### 2.11. Statistical Analysis

Parametric data were analyzed using GraphPad Prism 8 software and displayed as the means ± standard errors of the means (SEM). Significant differences between multiple groups were analyzed using one-way ANOVA, and *P* < 0.05 was considered statistically significant.

## 3. Results

### 3.1. Induction and Transplantation of MSCs

**Figure 2:**
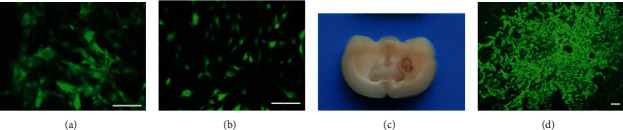
Induction and transplantation of MSCs. (a) Cultured MSCs labeled with GFP. Scale bar = 100 *μ*m. (b) MSCs induced by neuronal induction medium. Scale bar = 100 *μ*m. (c) The hemorrhagic area in the basal ganglia of the ICH model. (d) Transplanted MSC-dNLCs/GFP in the brains of ICH rats. Scale bar = 100 *μ*m.

MSCs labeled with GFP grew in a cluster shape and presented green fluorescence under a fluorescence microscope (Figure 2(a)). After induction with neuronal induction medium, MSC-dNLCs showed changes resembling neuron-like cells (Figure 2(b)). The hemorrhagic area was still seen in the basal ganglia region in the coronal incision (Figure 2(c)). Transplanted MSC-dNLCs were observed in rats from the MSC-dNLCs and MSC-dNLCs+EA groups (Figure 2(d)).

### 3.2. The Combined Treatment Reduced the Number of Blood Vessels and Attenuated Brain Edema

**Figure 3:**
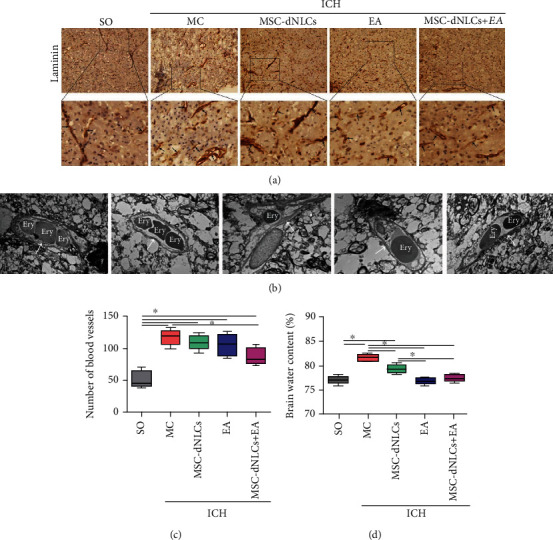
The combined treatment reduced the number of blood vessels and attenuated brain edema in rats with ICH. (a) Images of laminin immunohistochemistry in the ipsilateral striatum of rats. The black arrows show blood vessels. Scale bar = 100 *μ*m. (b) Electron photomicrographs of blood vessels in the peripheral area of hemorrhagic foci. The white arrows show capillaries, and ery indicates erythrocytes. Scale bar = 5 *μ*m. (c) The number of blood vessels in different groups (*n* = 5 rats per group; ^∗^*P* < 0.05). (d) The brain water content of brain samples from different groups (*n* = 5 rats per group; ^∗^*P* < 0.05).

Immunohistochemical staining for laminin was used to observe blood vessels. The number of blood vessels increased obviously in rats after ICH (*P* < 0.05), and the combined treatment significantly reduced the number of blood vessels (30.32 ± 9.71%) compared with the MC group (*P* < 0.05), but the numbers of blood vessels in the three treatment groups were not significantly different (*P* > 0.05) (Figures 3(a) and 3(c)). TEM showed that although the tissue structure around blood vessels was loose in ICH rats, the vascular structure of each group was intact (Figure 3(b)). The BWC increased significantly in rats after ICH (*P* < 0.05) but was decreased in the EA, MSC-dNLCs, and MSC-dNLCs+EA groups compared with the MC group (*P* < 0.05) (Figure 3(d)). Moreover, EA stimulation and the combined treatment reduced the BWC to a greater extent than MSC-dNLC transplantation in ICH rats (Figure 3(d)).

### 3.3. The Combined Treatment Increased the Levels of the MAP2 and GALC Proteins and Decreased the Level of the GFAP Protein

**Figure 4:**
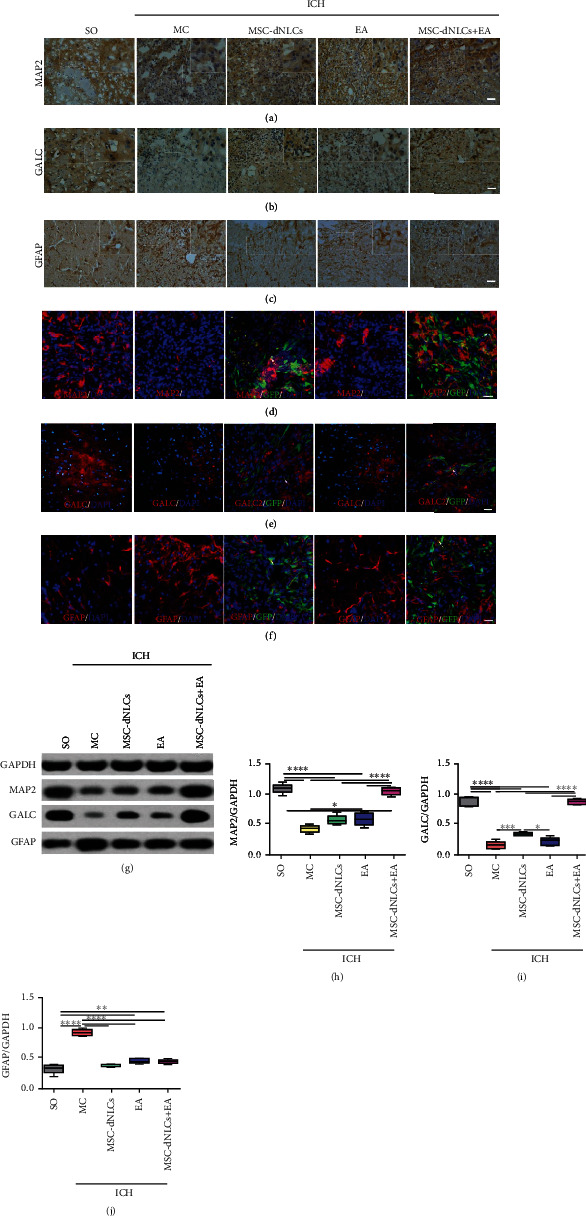
The expression of the MAP2, GALC, and GFAP proteins in different groups was detected using immunohistochemical staining and western blotting. (a–c) Representative images of MAP2 (a), GALC (b), and GFAP (c) immunohistochemical staining in the ipsilateral striatum of animals from different groups. Scale bar = 100 *μ*m. (d–f) Representative images of MAP2 (d), GALC (e), and GFAP (f) immunofluorescence staining in the ipsilateral striatum of rats from different groups. Scale bar = 100 *μ*m. (g–j) Representative results (g) and quantitative analyses (h–j) of western blotting showed the relative expression of the MAP2, GALC, and GFAP proteins in the ipsilateral striatum of rats from different groups (*n* = 5 animals per group; ^∗^*P* < 0.05, ^∗∗^*P* < 0.01, ^∗∗∗^*P* < 0.001, and ^∗∗∗∗^*P* < 0.0001).

The expression of the MAP2 (neuron marker), GALC (oligodendrocyte marker), and GFAP (astrocyte marker) proteins was detected using immunohistochemical staining and western blotting. Figures 4(a)–4(c) show the distribution of MAP2-, GALC-, and GFAP-positive cells. Immunofluorescence staining revealed that a few of the transplanted cells (GFP-positive cells) coexpressed MAP2, GALC, and GFAP in both the MSC-dNLCs and MSC-dNLCs+EA groups (Figures 4(d)–4(f)). The results of western blotting showed that the levels of the MAP2 and GALC proteins decreased, but the level of the GFAP protein was increased in rats after ICH (*P* < 0.05, Figures 4(g)–4(j)). Compared with the MC group, the expression level of the MAP2 protein was increased in the EA and MSC-dNLCs+EA groups, and the level of the GALC protein was increased in the MSC-dNLCs and MSC-dNLCs+EA groups, but the level of the GFAP protein was decreased in the EA, MSC-dNLCs, and MSC-dNLCs+EA groups (*P* < 0.05, Figures 4(g)–4(j)). Moreover, levels of the MAP2 and GALC proteins increased significantly in the MSC-dNLCs+EA group compared with the EA and MSC-dNLCs groups (*P* < 0.05, Figures 4(g)–4(i)).

### 3.4. The Combined Treatment Increased the Levels of Synapse-Related Proteins

**Figure 5:**
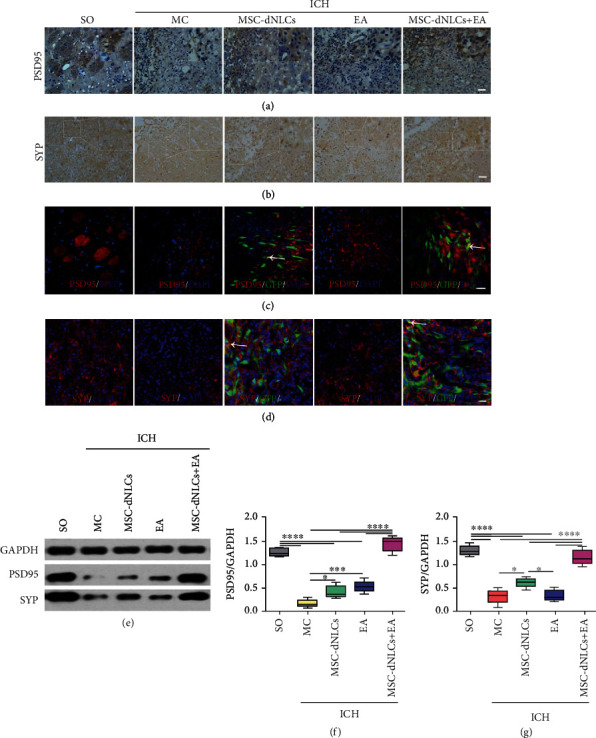
The expression of the PSD95 and SYP proteins in different groups. (a, b) Representative images of PSD95 (a) and SYP (b) immunohistochemical staining in the ipsilateral striatum of rats from different groups. Scale bar = PSD96. (c, d) Representative images of PSD95 (c) and SYP (d) immunofluorescence staining in the ipsilateral striatum of rats from different groups. Scale bar = 100 *μ*m. (e–g) Representative results (e) and quantitative analyses (f, g) of western blotting showed the relative expression of the PSD95 and SYP proteins in the ipsilateral striatum of rats from different groups (*n* = 5 animals per group; ^∗^*P* < 0.05, ^∗∗∗^*P* < 0.001, and ^∗∗∗∗^*P* < 0.0001).

Figures 5(a) and 5(b) show the distribution of PSD95 and SYP immunopositive products. In addition, the histological evaluation of transplanted MSC-dNLCs confirmed the coexpression of GFP and PSD95 or SYP in the MSC-dNLCs and MSC-dNLCs+EA groups (Figures 5(c) and 5(d)). Western blotting analyses revealed decreased levels of the PSD95 and SYP proteins in rats after ICH (*P* < 0.05, Figures 5(e)–5(g)). PSD95 expression increased in the EA, MSC-dNLCs, and MSC-dNLCs+EA groups, and SYP expression increased in the MSC-dNLCs and MSC-dNLCs+EA groups compared with the MC group (*P* < 0.05, Figures 5(e)–5(g)). Furthermore, the combined treatment obviously increased the levels of the PSD95 and SYP proteins compared with the single treatment (*P* < 0.05, Figures 5(e)–5(g)).

### 3.5. The Combined Treatment Increased the Number of Synapses

**Figure 6:**
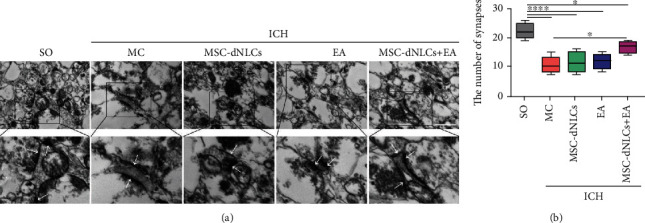
The combined treatment increased the number of synapses in rats with ICH. (a) Electron photomicrographs of synapses in the ipsilateral striatum of brain tissues from different groups. The white arrows show synapses. Scale bar = 1 *μ*m. (b) The number of synapses in the ipsilateral striatum (100 *μ*m^2^ area) (*n* = 5 rats per group; ^∗^*P* < 0.05 and ^∗∗∗∗^*P* < 0.0001).

The synapse density was calculated to quantitatively investigate synaptic degradation, and the results showed a reduced number of synapses (per 100 *μ*m^2^) in rats after ICH (*P* < 0.05). However, a significant difference in the number of synapses was not observed among the EA, MSC-dNLCs, and MSC-dNLCs+EA groups (Figure 6). In addition, the number of synapses increased in only the MSC-dNLCs+EA group compared with that in the MC group (*P* < 0.05, Figure 6).

### 3.6. The Combined Treatment Activated mTOR and p70S6K

**Figure 7:**
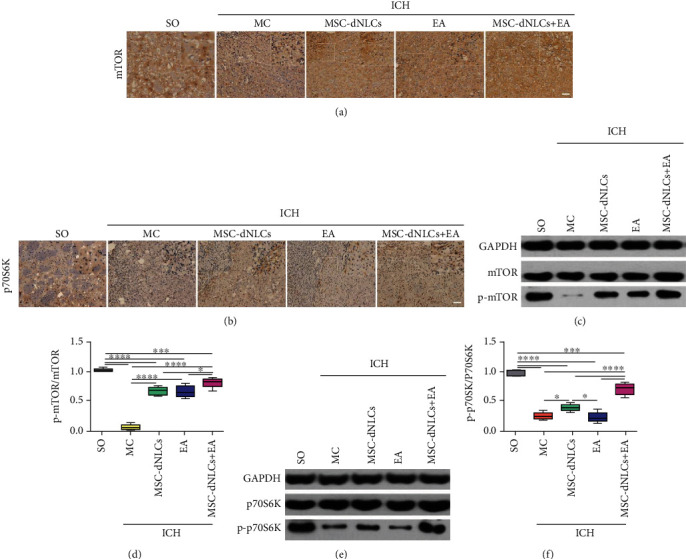
The levels of total and phosphorylated mTOR and p70S6K proteins in different groups. (a, b) Representative images of mTOR (a) and p70S6K (b) immunohistochemistry in the ipsilateral striatum of brain tissues from different groups. Scale bar = 100 *μ*m. (c, d) Representative results (c) and quantitative analyses (d) of western blotting showed the level of the phosphorylated mTOR protein in different groups (*n* = 5 animals per group; ^∗^*P* < 0.05, ^∗∗∗^*P* < 0.001, and ^∗∗∗∗^*P* < 0.0001). (e, f) Representative results (e) and quantitative analyses (f) of western blotting showed the level of the phosphorylated p70S6K protein in different groups (*n* = 5 animals per group; ^∗^*P* < 0.05, ^∗∗∗^*P* < 0.001, and ^∗∗∗∗^*P* < 0.0001).

Immunohistochemical staining showed that mTOR and p70S6K proteins were expressed in all groups (Figures 7(a) and 7(b)). The western blot results showed significantly decreased levels of the phosphorylated mTOR and p70S6K proteins in rats after ICH (*P* < 0.05, Figures 7(c)–7(f)). EA stimulation increased the level of the phosphorylated mTOR protein, and MSC-dNLC transplantation increased the levels of the phosphorylated mTOR and p70S6K proteins (*P* < 0.05, Figures 7(c)–7(f)). Furthermore, compared with the MSC-dNLCs and EA groups, the levels of the phosphorylated mTOR and p70S6K proteins were obviously increased in the MSC-dNLCs+EA group (*P* < 0.05, Figures 7(c)–7(f)).

## 4. Discussion

The present study investigated the effect of the combination of MSC-dNLC transplantation and EA stimulation on synaptic plasticity and its molecular mechanisms in ICH rats. For this purpose, we evaluated cell survival, the number of synapses, the levels of synapse-associated proteins, and the levels of the phosphorylated mTOR and p70S6K proteins. The results suggested that the combined treatment exerts a synergistic effect on improving synaptic plasticity through mTOR/p70S6K signaling.

After ICH, brain edema occurs early, with a substantial increase of approximately 75% of its maximum volume during the first 24 hours, and then continues to develop over an extended period of days to nearly 2 weeks [2, 37, 38]. Brain edema is considered a radiological marker and contributes to poor outcomes in patients with ICH due to secondary injury after ICH [38–40]. In this study, our results showed that the three different treatments all reduced the BWC in ICH rats, and the BWC decreased more significantly in the MSC-dNLCs+EA group than in the MSC-dNLCs group. Thus, EA stimulation further alleviates brain edema after MSC-dNLC transplantation. Laminin, one of the primary functional components in basement membranes of blood vessels in most tissues [41–43], has been used to visualize blood vessels through immunohistochemical staining. Laminin overexpression, which is one of the consequences of vasogenic edema, may promote the migration of newly generated vessels to repair blood–brain barrier (BBB) disruption [44]. Loss of laminin aggravates BBB damage by regulating brain water homeostasis in ICH mice [45]. In this experiment, we observed an increased number of blood vessels in the MC, EA, MSC-dNLCs, and MSC-dNLCs+EA groups compared with the SO group, and the vascular structure was intact. Interestingly, the combined treatment reduced the number of blood vessels in ICH rats. The combined treatment inhibits hyperplasia of blood vessels, and a proper number of blood vessels might decrease the BWC in rats with ICH.

ICH may cause massive cell death, including glial cells and neurons [46, 47], in the brain through many mechanisms, such as inflammation [48], oxidative stress [49], and cytotoxicity [50]. Oligodendrocyte and neuron death are associated with demyelination and neurological dysfunction [51, 52]. Reactive astrogliosis, a pathological change associated with CNS injury, potentially promotes brain repair and reduces neurological impairment [53]. Reactive astrocytes exert beneficial effects by secreting neurotrophic substances that protect neurons at early stages [54]. However, excessive proliferation of reactive astrocytes leads to glial scar formation, which definitely impairs axon growth and neural network reconstruction during later periods [53, 54]. Previous studies on MSC transplantation in stroke have implied that MSCs improve neurological recovery, reduce apoptosis, inhibit scar formation, and enhance reactive astrocyte- and oligodendrocyte-related axonal remodeling [55, 56]. MSCs and EA treatment could improve the expression levels of trophic factors such as brain-derived neurotrophic factor (BDNF), neurotrophin-4 (NT4), and vascular endothelial growth factor (VEGF) in ischemic stroke, which are all very helpful for brain tissue regeneration [26]. MSC transplantation combination with EA treatment increased the number of neurofilament-200 positive fibers and BDA-labeled descending corticospinal tract (CST) axons in the lesion site of the injured spinal cord compared to MSC transplantation or EA treatment alone and downregulated expressions of GFAP and chondroitin sulfate proteoglycan (CSPG) proteins compared with the operated control, which inhibited axonal degeneration as well as promoted axonal regeneration [24]. In the present study, EA stimulation increased the survival of neurons, and MSC-dNLC transplantation increased the survival of oligodendrocytes, and combination of the two also inhibited the proliferation of astrocytes. Furthermore, some of the transplanted cells expressed markers of neurons and glial cells. Notably, the combined treatment exerted a synergistic effect on promoting the survival of neurons and oligodendrocytes, which helped to improve the structure of the brain tissue.

The effects of the combined treatment on synaptic plasticity in the focal area were indicated by the increased levels of synaptic structural molecules. PSD-95, a major component of glutamatergic excitatory synapses, is a scaffolding protein that modulates the synaptic localization of many adhesion molecules, channels, receptors, and signaling proteins [57, 58]. Previous studies have documented important roles for PSD95 in the formation and maintenance of synapses, and current reports have focused on PSD95 and its molecular mechanisms underlying synaptic maturation and plasticity [58, 59]. SYP has been authenticated as one of the first nerve terminal proteins [60] and performs essential functions in synaptic plasticity [61]. Some studies showed that coculture with MSCs increases the expression of synaptic density markers (such as PSD95 and SYP) in A*β*42-treated primary hippocampal neurons, and EA increases the levels of the SYP, PSD-95, and GAP-43 proteins, enhanced synaptic structural plasticity, and improved behavioral performance in rats exposed to chronic unpredictable mild stress [62, 63]. Based on our data, MSC-dNLC transplantation increased the expression of the PSD95 and SYP proteins, a few transplanted cells also expressed PSD95 and SYP proteins, and EA further increased the expression of the two proteins in ICH rats transplanted with MSC-dNLCs. Interestingly, the combination therapy also increased the number of synapses. Therefore, our results indicated that the combined therapy may contribute to improving synapse plasticity in rats with ICH.

The mTOR/p70S6K pathway is involved in stroke [32, 64]. mTOR, a serine/threonine protein kinase, is a central regulator of protein, lipid, and nucleotide synthesis, autophagy, cell survival, and proliferation [32, 34, 65]. p70S6K is one of the downstream targets of mTOR [66, 67]. The p-mTOR and p-p70S6k proteins are regarded as markers of mTOR activity [68]. However, controversy exists regarding whether mTOR activity is beneficial or detrimental to the damaged brain. Its activity increases when the injured brain is protected by some neuroprotectants, including alpha-lipoic acid, melatonin, and silibinin [64, 69, 70], suggesting that activated mTOR is beneficial for brain injury. In contrast, studies have also reported that mTOR activity is detrimental to cognitive function and that minocycline prevents cognitive deficits by inhibiting mTOR signaling, increasing the autophagy process, and increasing the expression of pre- and postsynaptic proteins (SYP and PSD95) in rats after acute ischemic stroke [71]. Our results show that MSC-dNLC transplantation increased the levels of the phosphorylated mTOR and p70S6k proteins and that EA further increased mTOR activity in ICH rats after MSC-dNLC transplantation. Some researchers have reported that EA improves learning and memory functions by upregulating the expression of mTOR in rats with vascular dementia or cerebral ischemia/reperfusion [72, 73], and MSCs affect mTOR signaling through paracrine effects or exosomes [74–76].

Recent studies have provided evidence that the neuroprotective effects of mTOR on stroke may be due to its ability to increase PSD95 and GAP-43 protein levels or promote neuronal structural stability in peri-infarct regions [77, 78]. Li et al. reported that the mTOR pathway activated by ketamine increases the levels of synaptic signaling proteins (synapsin I, PSD95, and GluR1) and increases the number and function of new synapses in the prefrontal cortex of rat models of depression [33]. As mentioned above, the combined therapy increased the expression of synapse-associated proteins (SYP and PSD95), and the results suggest that the protective effects of the combined therapy on mTOR may be related to its ability to increase synaptic plasticity in the hemorrhagic stroke.

The present study has some limitations. First, the observations of mTOR/p70S6K signaling and its regulation were limited to the ICH rat model. This information should be carefully considered when using the results to discuss the role of the mTOR pathway in neurological disease. Second, we did not construct mTOR and p70S6K viruses to obtain further proof. mTOR/p70S6K signaling is an interesting target in hemorrhagic stroke that requires further investigation in subsequent research.

In summary, despite the shortcomings of this study, we provide the first evidence for the synergistic effects of a combined treatment consisting of MSC-dNLC transplantation and EA stimulation on improving synaptic plasticity, which may be associated with activated mTOR/p70S6K signaling in rats with ICH. These findings not only provide new data for the neuroprotective effect of a combined treatment consisting of MSC-dNLC transplantation and EA stimulation but also supply positive insights that will improve our understanding of the underlying molecular mechanisms and help to develop more precise therapeutic drugs and treatments for alleviating the outcomes of ICH.

## Data Availability

The data that support the findings of this study are available from the corresponding author upon reasonable request.
